# Factors associated with severe acute respiratory syndrome coronavirus-2 infection in Hohoe Municipality, Ghana: A case-control study

**DOI:** 10.1371/journal.pone.0332561

**Published:** 2026-07-24

**Authors:** Sadat Ibrahim, Yussif Yakubu, Kwaku Appiagyei, Adjato Franklin Duncan Sylvester, Yahuza Sabit Tanko, Frank Baiden

**Affiliations:** 1 Department of Epidemiology and Biostatistics, Fred N. Binka School of Public Health, University of Health and Allied Sciences, Ho, Volta Region, Ghana; 2 KNUST-International Vaccine Institute Collaborative Center, Asante Akim Agogo, Ashanti Region, Ghana; UG NMIMR: University of Ghana Noguchi Memorial Institute for Medical Research, GHANA

## Abstract

Severe acute respiratory syndrome coronavirus-2 (SARS-CoV-2) infection was a major public health challenge globally and in Ghana. To prepare better for future pandemics, an evidence-based understanding of the determinants of coronavirus disease is essential to inform public health guidelines and surveillance. Thus, we identified the factors for SARS-CoV-2 infection in Hohoe Municipality. We conducted a facility-based, sex and age-matched (1:2) case-control study. Cases were persons with a laboratory-confirmed SARS-CoV-2 infection by Reverse Transcription Polymerase Chain Reaction (RT-PCR) or rapid antigen test, while controls tested negative with the same techniques. Data on sociodemographic, clinical, and exposure-related factors were collected through structured interviews. We employed a conditional regression model to establish the factors independently associated with SARS-CoV-2 infection using Stata version 17.0. All statistical tests were two-sided, and a p-value <0.05 in the final multivariate logistic model was considered statistically significant. A total of 234 participants were enrolled (78 cases, 156 controls). The mean age of the cases and controls was 39.7 (±14.6) and 39.4 (±14.4) years, respectively. Moderate/high levels of social interaction increased the odds of infection (aOR=3.00, 95% CI:1.05–8.56, p = 0.040). Having no underlying health condition (aOR=0.25, 95% CI:0.09–0.65, p = 0.004) and regular physical activity or exercise (aOR=0.18, 95% CI:0.04–0.70, p = 0.014) reduced the risk of infection. Public health interventions should therefore prioritize strengthening community awareness about the risks of close social interactions and the benefits of healthy lifestyles, including regular physical activity.

## Introduction

The novel Severe Acute Respiratory Syndrome Coronavirus-2 (SARS-CoV-2) was the etiologic agent of the Coronavirus Disease 2019 (COVID-19) pandemic. It caused widespread infection, morbidity, and mortality from late 2019 and created a global health emergency [1]. On January 30, 2020, the World Health Organization (WHO) declared COVID-19 a public health emergency of international concern, and on March 11, 2020, a pandemic [1,2]. Ghana’s index case was confirmed on March 12, 2020 [3]. In the African context, particularly in sub-Saharan Africa, the epidemiology of COVID-19 was shaped by unique demographic, socio-economic, and health system factors. Although the region reported comparatively lower case fatality rates than many high-income settings, limited testing capacity and under-detection contributed to uncertainty in the true burden of the disease [4,5]. Evidence from the region suggests that over 80% of infections were either asymptomatic or mild [2], indicating a substantial proportion of undetected cases within communities. Clinical presentations ranged from asymptomatic to severe or critical illness [6], complicating case identification and surveillance efforts. This, coupled with the high proportion of pre-symptomatic and asymptomatic infections, likely facilitated undetected community transmission, as infected individuals could spread the virus without being readily identified. This made pre-symptomatic and asymptomatic patients a hidden potential source of transmission. Direct or indirect contact with respiratory aerosols, droplets or other bodily fluids via contaminated surfaces was the known predominant mode of transmission [7,8].

The Centers for Disease Control and Prevention defined risk factors as a myriad of factors that could facilitate an individual’s exposure, or response to a causative agent and increase susceptibility, or predispose to a disease or infection [9]. Although COVID-19 is primarily transmitted through exposure to the infectious agent, several factors drive susceptibility and disease severity. These include demographic characteristics (e.g., age), pre-existing comorbidities (such as hypertension, diabetes mellitus, cardiovascular diseases, and chronic respiratory conditions), and clinical characteristics, including symptom presentation and disease severity at diagnosis [10,11]. Moreover, environmental factors like temperature and humidity, health system organization and policy, politico-economic situation, large gatherings, and international travel could substantially drive community transmission [12–14].

Even with the abundance of epidemiological data produced during the pandemic, significant knowledge gaps persist regarding the specific factors associated with SARS-CoV-2 infection in rural and peri-urban African communities. Thus far, no prior studies have established evidence on the determinants of SARS-CoV-2 infection within Hohoe Municipality. We identified the factors for SARS-CoV-2 infection in Hohoe Municipality. We seek to provide evidence-based insights that can guide surveillance and local public health interventions and contribute to the broader understanding of SARS-CoV-2 infection epidemiology in the study setting and Ghana by extension. The results contribute to the larger body of global health evidence on SARS-CoV-2 disease. Also, it offers implications for informing public health efforts, strengthening surveillance and pandemic preparedness and response to emerging and reemerging infectious illnesses.

## Methods

### Study design

We adopted a matched case-control design to identify the factors for SARS-CoV-2 infection. To eliminate confounders, the controls were individually matched (1 case: 2 controls) to the cases, in which the enrolled controls were similar to the cases in age (±5 years) and sex.

### Study site

**Fig 1:**
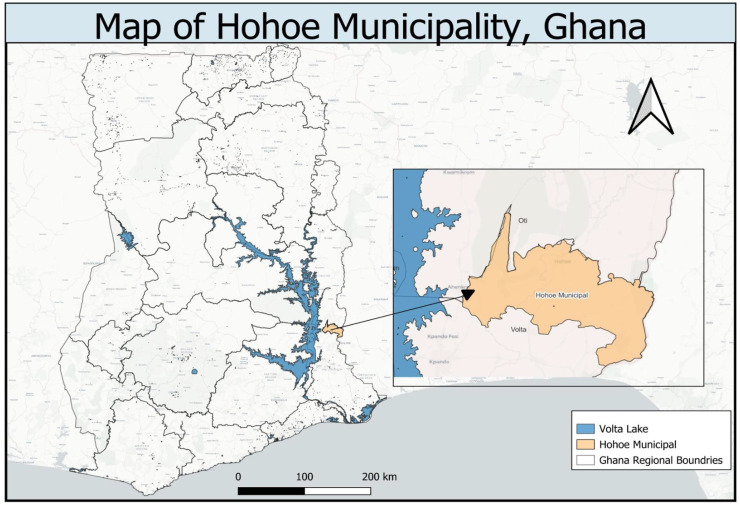
Map of Hohoe Municipality. The map depicts the geographical location of Hohoe Municipality in the Volta Region of Ghana, where a case-control study investigating factors associated with SARS-CoV-2 infection was conducted between March 2020 and December 2021.

The study was conducted in Hohoe Municipality, located in the Volta Region of Ghana in West Africa. The municipality lies in the eastern part of the country and shares an international boundary with the Republic of Togo. Hohoe serves as the administrative capital of the municipality. The municipality has an estimated population of 114,472, representing approximately 6.8% of the total population of the Volta Region [15]. Females constitute 52.1% of the population, while males account for 47.9% [15]. Slightly over half of the population resides in urban areas, distributed across several towns and settlements. Healthcare delivery in the municipality is organized into sub-districts and includes a network of primary and secondary health facilities. The Volta Regional Hospital, located in Hohoe, is the main referral facility and provides specialized care to residents within the municipality and surrounding districts. Additional health services are delivered through community-based health planning and services compounds and health centres, which support primary healthcare and public health interventions, including infectious disease surveillance and response Fig 1.

### Study population

The study population comprised residents of Hohoe Municipality who were tested for SARS-CoV-2 infection using Reverse Transcription Polymerase Chain Reaction (RT-PCR) or rapid antigen tests between March 2020 and December 2021. Individuals with a laboratory-confirmed positive result by RT-PCR or rapid antigen test were classified as cases, while those with negative results using the same methods were considered eligible controls.

Inclusion criteria were individuals aged 18 years or older who had a documented SARS-CoV-2 test result (positive or negative) by RT-PCR or rapid antigen testing within the specified period. Exclusion criteria included individuals who were diagnosed using other testing methods, such as serological (antibody-based) assays or clinical and radiological assessments without laboratory confirmation, due to their limited specificity for detecting active infection. Additionally, individuals who had previously tested positive for SARS-CoV-2 and subsequently tested positive again were excluded as potential cases of reinfection. Reinfection status was determined based on the municipal SARS-CoV-2 surveillance line list and clinical records, which documented prior positive test results followed by a subsequent positive result after recovery. Furthermore, individuals who tested positive beyond December 2021, were not resident in the municipality at the time of data collection, had severe illness or cognitive impairment that limited their ability to respond, or had incomplete or missing key information in the surveillance line list were ineligible for inclusion.

### Sample size

A priori sample size calculation was performed using the Fleiss with Continuity Correction approach for case-control studies [16]. The calculation assumed a statistical power of 80% and a two-sided confidence level of 95% (Z = 1.96). A hypothesized odds ratio of 2.35 was specified as the minimum detectable effect size, with an estimated exposure prevalence of 40% among controls, based on findings from a previous COVID-19 study conducted in Ghana [6]. Based on these assumptions and a case-to-control ratio of 1:2, the required sample size for the study was 237 (79 cases, 158 controls).

### Sampling method

We employed systematic sampling to recruit cases using a line list of the cases as a sample frame. The line list was obtained from the Hohoe Municipal Health Directorate surveillance database and comprised laboratory-confirmed SARS-CoV-2 cases identified through routine surveillance of symptomatic and asymptomatic individuals. After applying the study eligibility criteria, 245 laboratory-confirmed SARS-CoV-2 cases constituted the case sampling frame, from which 79 cases were selected using systematic sampling to achieve the required sample size. The sampling interval was determined by dividing the total number of eligible cases (245) by the required sample size (79), yielding a sampling interval of approximately 3. A random starting point was selected from the first three eligible cases on the sampling frame, after which every third eligible case was selected until the required sample size was attained.

For the controls, individuals who tested negative for SARS-CoV-2 within the same surveillance database and met the eligibility criteria were considered. Controls were randomly selected using simple random sampling and were individually matched to cases in a ratio of 1:2 based on age (±5 years) and sex.

### Data collection procedure

Data were collected between September 2 and October 28, 2023 using a structured questionnaire administered through face-to-face interviews and captured electronically via KoboCollect. We identified participants from the municipal SARS-CoV-2 surveillance line list, which provided verified test dates and baseline information from March 2020 to December 2021. To address the approximately three-year recall period, we ensured that the questionnaire focused on major, easily recallable exposures (e.g., travel, social gatherings, healthcare visits, and underlying conditions). All exposure variables were anchored to the 14 days preceding the SARS-CoV-2 test date, which was verified from surveillance records and used to guide participant recall. Moreover, interviewers cross-checked selected responses against available surveillance and clinical records where feasible.

### Study variables

Data were collected on sociodemographic, clinical, and exposure-related variables. Sociodemographic characteristics included age, sex, educational level, occupation, marital status, income level, ethnicity, and place of residence (urban/rural). Additional household-level variables, such as household residence type (e.g., compound house, self-contained housing), National Health Insurance Scheme (NHIS) status (insured/uninsured), and number of children in the household before SARS-CoV-2 testing were also obtained. Clinical information and SARS-CoV-2-related contact history, exposure patterns, and outcomes were collected, with the reference period for both cases and controls defined as the 14 days preceding SARS-CoV-2 testing. Exposure-related variables were operationally defined before analysis. Close contact with a confirmed or suspected COVID-19 case referred to being within approximately one meter of an infected or suspected individual for at least 15 minutes or having direct physical contact during the 14 days preceding testing. Contact with a generally ill person referred to interaction with any person presenting symptoms suggestive of an infectious illness during the same period. Level of frequent social interaction was categorized based on self-reported daily interpersonal interactions and crowd exposure patterns: low crowding referred to limited routine interactions with few individuals and avoidance of crowded settings, whereas moderate/high crowding referred to regular interactions with larger groups, frequent attendance at social activities, or repeated exposure to crowded environments. Possible place of exposure referred to the location where participants believed they had the highest likelihood of encountering infection. Furthermore, self-reported behavioral risk information was obtained, including smoking status (current smoker/non-smoker) and non-smoking substance use, including alcohol consumption and use of other substances such as tramadol, inhalants or cocaine.

### Statistical analyses

Data collated on KoboCollect was exported to Microsoft Excel format. We then checked and resolved all discrepant data before formal analyses using Stata version 17.0 statistical software. Categorical variables were described using frequencies and percentages, and mean (standard deviation) was computed for continuous variables. Given the individually age- and sex-matched (1:2) case-control design, associations between potential risk factors and SARS-CoV-2 infection were first explored using univariate conditional logistic regression, reporting crude odds ratios (cORs) with 95% confidence intervals (CIs). Variables with p-values <0.25 in the univariate analysis were considered for inclusion in a multivariable conditional logistic regression model. Adjusted odds ratios (aORs) with 95% CIs were then estimated to identify factors independently associated with SARS-CoV-2 infection. All statistical tests were two-sided, and statistical significance was set at p-value <0.05 in the final model.

### Ethical considerations

The study protocol received ethical approval from the University of Health and Allied Sciences Research Ethics Committee (UHAS-RECA.8[88]22–23) and the Ghana Health Service Ethics Review Committee (GHS-ERC:029/06/23). Participants were provided with comprehensive information in their preferred language and gave voluntary informed consent prior to enrolment. No personally identifiable information was collected; instead, responses were assigned unique codes to ensure anonymity. All data were securely stored in password-protected electronic files and kept in a restricted-access environment, with access limited to authorized research personnel to safeguard sensitive information. The study posed no risk to participants, upheld strict confidentiality, and adhered to the ethical principles of the Declaration of Helsinki (1964).

## Results

### Participant selection and classification by SARS-CoV-2 testing method and outcome

**Fig 2:**
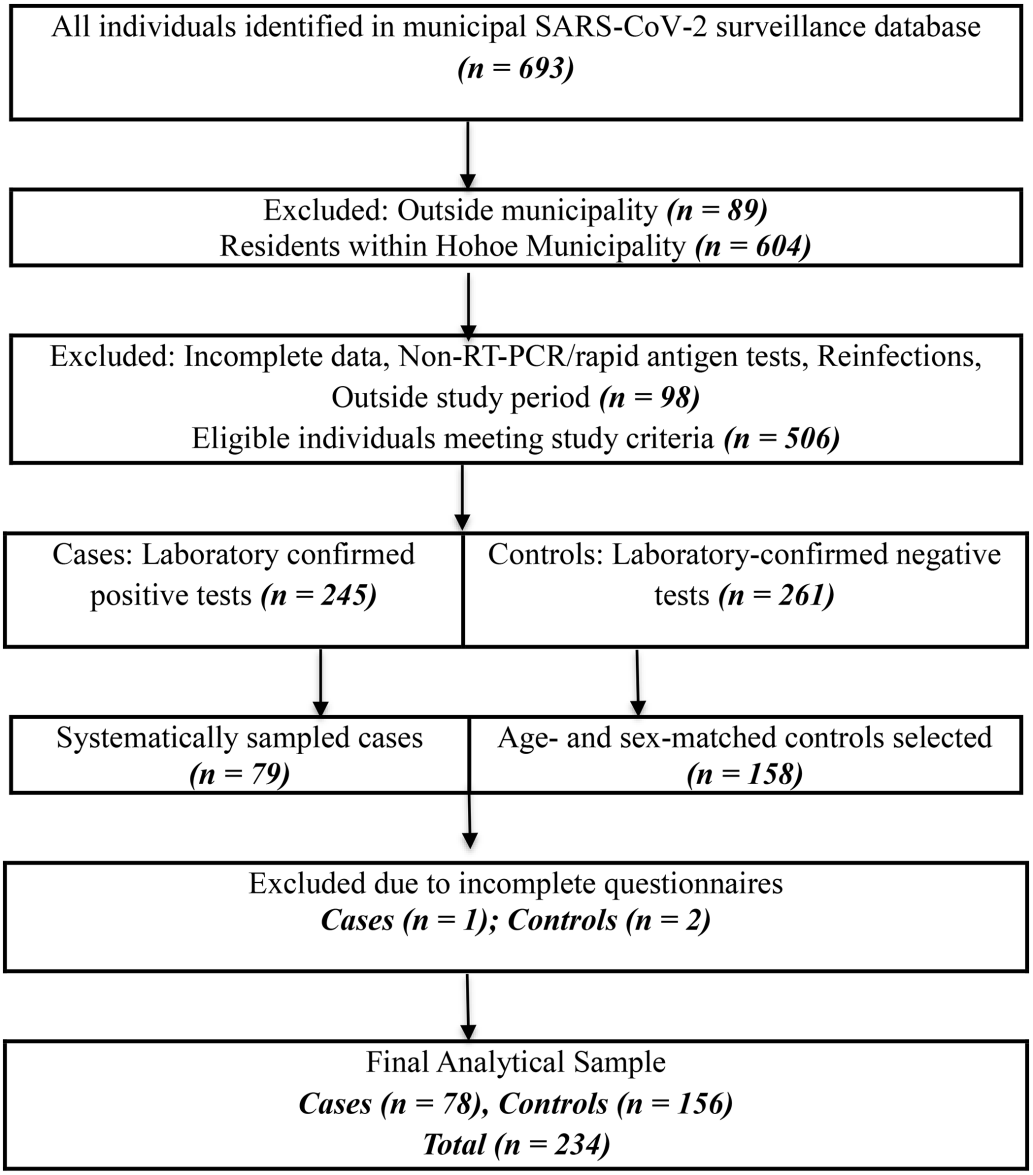
Participant selection flowchart and case-control classification for SARS-CoV-2 testing for the study. Flow diagram illustrating participant selection from the Hohoe Municipal SARS-CoV-2 surveillance database. Individuals were screened for eligibility, excluded according to predefined criteria, and sampled to obtain the final analytical population of 78 cases and 156 age- and sex-matched controls (n = 234).

Fig 2 depicts the selection and classification of participants based on SARS-CoV-2 testing method and outcomes in Hohoe Municipality between March 2020 and December 2021. A total of 693 individuals were initially identified from the Hohoe Municipal SARS-CoV-2 surveillance database, of whom 89 were excluded for residing outside the municipality. Of the 506 remaining eligible individuals, 245 had laboratory-confirmed SARS-CoV-2 infection and constituted the case sampling frame, while 261 individuals had negative test results and constituted the control sampling frame. Cases with suspected reinfection, individuals with incomplete records, and those diagnosed using non-approved methods were excluded before the final case and control sampling frames were established.

Systematic sampling was used to select 79 cases from the case sampling frame, while controls were selected by simple random sampling and matched individually to cases according to age (±5 years) and sex in a ratio of 1:2. Following recruitment and completion of data collection, one case and two matched controls were excluded because of incomplete questionnaire responses, resulting in a final analytical sample of 78 cases and 156 controls (n = 234).

### Sociodemographic characteristics of cases and controls

Data were gathered from 234 respondents, and the mean ages of the cases and controls were 39.7 (±14.6) and 39.4 (±14.4) years, respectively, with an overall mean age of 39.5 (±14.5) years (Table 1). Urban residence was higher among cases 62 (79.5%) than among controls 94 (60.3%). Less than half of the cases 21 (26.9%) were asymptomatic as against the controls 89 (57.0%). Of the cases, less than half 33 (42.3%) had been prediagnosed with a health condition compared to the controls 108 (69.2%). The proportion of cases with hypertension and diabetes was 23 (29.5%) and 18 (23.1%), respectively, versus hypertension 23 (14.7%) and diabetes 21 (13.5%) in the control arm.

**Table 1:** Sociodemographic Characteristics of Cases and Control.

Variable	Case (78) *n* (%)	Control (156) *n* (%)	Total (234) *n* (%)
**Mean age (±SD)**	39.7 (14.6)	39.4 (14.4)	39.5 (14.5)
**Age**			
≤ 30	28 (35.9)	53 (34.0)	81 (34.6)
≥ 31	50 (64.1)	103 (66.0)	153 (65.4)
**Sex**			
Male	40 (51.3)	80 (51.3)	120 (51.3)
Female	38 (48.7)	76 (48.7)	114 (48.7)
**Highest educational level**			
None	5 (6.4)	15 (9.6)	20 (8.5)
Primary	1 (1.3)	21 (13.5)	22 (9.4)
Junior High School	11 (14.1)	33 (21.1)	44 (18.8)
Senior High School	21 (26.9)	31 (19.9)	52 (22.2)
Tertiary/post-secondary	40 (51.3)	56 (35.9)	96 (41.0)
**Occupation**			
Unemployed	21 (26.9)	43 (27.6)	64 (27.4)
Formal	28 (35.9)	35 (22.4)	63 (26.9)
Informal/Self-employed	29 (37.2)	78 (50.0)	107 (45.7)
**Marital status**			
Single	29 (37.2)	55 (35.3)	84 (35.9)
Married	47 (60.3)	83 (53.2)	130 (55.6)
Divorced/widowed	2 (2.6)	18 (11.5)	20 (8.5)
**Income level**			
High	7 (9.0)	18 (11.5)	25 (10.7)
Low	4 (4.1)	23 (14.7)	27 (11.5)
Middle	67 (85.9)	115 (73.7)	182 (77.8)
**Residence**			
Rural	16 (20.5)	62 (39.7)	78 (33.3)
Urban	62 (79.5)	94 (60.3)	156 (66.7)
**Household residence type**			
Compound house setting	50 (64.1)	76 (48.7)	126 (53.8)
Private/self-contained setting	28 (35.9)	80 (51.3)	108 (46.2)
**NHIS status prior to testing**			
Active	63 (80.8)	107 (68.6)	170 (72.6)
Inactive	10 (12.8)	29 (18.6)	39 (16.7)
Not registered	5 (6.4)	20 (12.8)	25 (10.7)
**Number of children prior to testing**			
None	33 (42.3)	53 (34.0)	86 (36.7)
≥ 1	45 (57.7)	103 (66.0)	148 (63.3)
**Engaged in regular physical activities/exercised prior to testing**			
No	67 (85.9)	100 (64.1)	167 (71.4)
Yes	11 (14.1)	56 (35.9)	67 (28.6)
**COVID-19 symptom classification**			
Asymptomatic	21 (26.9)	89 (94.9%)	110 (47.0)
Symptomatic	57 (73.1)	67 (43.0)	124 (53.0)
**Ever been diagnosed with a health condition**			
No	45 (57.7)	48 (30.8)	93 (39.7)
Yes	33 (42.3)	108 (69.2)	141 (60.3)
**Hypertension**			
No	55 (70.5)	133 (85.3)	188 (80.3)
Yes	23 (29.5)	23 (14.7)	46 (19.7)
**Diabetes**			
No	60 (76.9)	135 (86.5)	195 (83.3)
Yes	18 (23.1)	21 (13.5)	39 (16.7)
**Smoking status prior to testing**			
Non-smoker	62 (79.5)	138 (88.5)	200 (85.5)
Smoker	16 (20.5)	18 (11.5)	34 (14.5)
**Non-smoking substance (alcohol, cocaine, tramadol, inhalants, codeine syrups) use prior to testing**			
**No**	51 (65.4)	131 (84.0)	182 (77.8)
Yes	27 (34.6)	25 (16.0)	52 (22.2)

### Contact history and disease exposure of cases and controls

Table 2 illustrates the contact history and disease exposure of the respondents. Among the cases, the majority 47 (60.3%) attended a gathering of less than ten people, and a greater 54 (69.2%) proportion travelled within Ghana. Most 65 (83.3%) of the cases had a moderate/high level of frequent social interactions compared to 82 (52.6%) of the controls.

**Table 2:** Contact History and Disease Exposure of Cases and Controls.

Variable	Case (78) *n* (%)	Control (156) *n* (%)	Total (234) *n* (%)
**Lived in a densely populated or crowded area prior to testing**			
No	55 (70.5)	123 (78.8)	178 (76.1)
Yes	23 (29.5)	33 (21.2)	56 (23.9)
**Attended a mass gathering prior to testing**			
No	43 (55.1)	95 (60.9)	138 (59.0)
Yes	35 (44.9)	61 (39.1)	96 (41.0)
**Attended a gathering of fewer than ten people prior to testing**			
No	31 (39.7)	92 (59.0)	123 (52.6)
Yes	47 (60.3)	64 (41.0)	111 (47.4)
**Travelled to a place within Ghana**			
No	24 (30.8)	98 (62.8)	122 (52.1)
Yes	54 (69.2)	58 (37.2)	112 (47.9)
**Travel location**			
Rural	14 (25.9)	25 (43.9)	39 (35.1)
Urban	40 (74.1)	32 (56.1)	72 (64.9)
**Visited a healthcare facility prior to testing**			
No	34 (43.6)	103 (66.0)	137 (58.6)
Yes	44 (56.4)	53 (34.0)	97 (41.4)
**Had close contact with a suspected or confirmed COVID-19 case prior to testing**			
No	63 (80.8)	146 (93.6)	209 (89.3)
Yes	15 (19.2)	10 (6.4)	25 (10.7)
**Had close contact with a generally ill person prior to testing**			
No	58 (74.4)	146 (93.6)	204 (87.2)
Yes	20 (25.6)	10 (6.4)	30 (12.8)
**Usual/most frequent means of transportation prior to testing**			
Private means	18 (23.1)	53 (34.0)	71 (30.3)
Public means	60 (76.9)	103 (66.0)	163 (69.7)
**Level of frequent social interactions prior to testing**			
Low crowding	13 (16.7)	82 (52.6)	95 (40.6)
Moderate/high crowding	65 (83.3)	74 (47.4)	139 (59.4)
**Place of most possible exposure**			
**Health facility**			
No	59 (75.6)	146 (93.9)	205 (87.6)
Yes	19 (24.4)	10 (6.4)	29 (12.4)
**Workplace**			
No	59 (75.6)	111 (71.2)	170 (72.6)
Yes	19 (24.4)	45 (28.8)	64 (27.4)
**Had access to PPE**			
No	30 (38.5)	56 (35.9)	86 (36.7)
Yes	48 (61.5)	100 (64.1)	148 (63.3)

### Univariate and conditional multivariate logistic regression analyses of severe acute respiratory syndrome coronavirus-2 infection risk factors

Univariate logistic regression analysis displayed in Table 3 revealed several factors statistically associated with SARS-CoV-2 infection. These included household residence type (cOR=0.55, 95% CI: 0.31–0.95, p = 0.032), symptom classification (cOR=3.57, 95% CI: 1.92–6.63, p < 0.001), number of children (cOR=0.33, 95% CI: 0.11–0.96, p = 0.042), physical activity (cOR=0.17, 95% CI: 0.06–0.45, p < 0.004), gathering of less than ten people (cOR=2.13, 95% CI: 1.22–3.71, p < 0.008), and level of social interaction (cOR=5.82, 95% CI: 2.80–12.11, p < 0.001). Additionally, a health facility as a possible place of exposure (cOR=4.85, 95% CI: 2.02–11.63, p < 0.001) showed an association. NHIS status (cOR=1.93, 95% CI: 1.00–3.74, p = 0.050) was borderline statistically significant.

**Table 3:** Univariate and multivariate logistic regression analyses of predictors for SARS-CoV-2 Infection.

Variable	cOR (95% CI)	*p-value*	aOR (95% CI)	*p-value*
**Residence**				
Rural	Ref.		Ref.	
Urban	**2.44 (1.30-4.60)**	**0.006**	1.16 (0.45-3.03)	0.758
**Household residence type**				
Compound house setting	Ref.		Ref.	
Private/self-contained	**0.55 (0.31-0.95)**	**0.032**	0.57 (0.25-1.30)	0.182
**NHIS status prior to testing**				
Inactive	Ref.		Ref.	
Active	**1.93 (1.00-3.74)**	**0.050**	1.51 (0.52-4.37)	0.447
**Symptom classification**				
Asymptomatic	Ref.		Ref.	
Symptomatic	**3.57 (1.92-6.63)**	**<0.001**	2.21 (0.90-5.41)	0.082
**Number of children had prior to testing**				
None	Ref.		Ref.	
≥ 1	**0.33 (0.11-0.96)**	**0.042**	0.21 (0.03-1.37)	0.103
**Diagnosed with underlying health condition(s) prior to testing**				
No	**0.25 (0.13-0.50)**	**<0.001**	**0.25 (0.09-0.65)**	**0.004**
Yes	Ref.		Ref.	
**Engaged in physical activities or exercises regularly prior to testing**				
No	Ref.		Ref.	
Yes	**0.17 (0.06-0.45)**	**<0.004**	**0.18 (0.04-0.70)**	**0.014**
**Attended gathering with less than ten people prior to testing**				
No	Ref.		Ref.	
Yes	**2.13 (1.22-3.71)**	**0.008**	2.13 (0.87-5.18)	0.097
**Travelled to another region, town or place prior to testing**				
No	Ref.		Ref.	
Yes	**3.42 (1.92-6.07)**	**<0.007**	2.17 (0.92-5.10)	0.076
**Visited a healthcare facility prior to testing**				
No	Ref.		Ref.	
Yes	**2.57 (1.44-4.58)**	**0.001**	1.86 (0.73-4.76)	0.195
**Had close contact with a suspected or confirmed COVID-19 case prior to testing**				
No	Ref.		Ref.	
Yes	**3.72 (1.50-9.24)**	**0.005**	0.64 (0.12-3.33)	0.598
**Had close contact with a generally ill person prior to testing**				
No	Ref.		Ref.	
Yes	**4.30 (1.95-9.46)**	**<0.001**	1.16 (0.30-4.43)	0.832
**Level of frequent social interactions prior to testing**				
Low crowding	Ref.		Ref.	
Moderate/high crowding	**5.82 (2.80-12.11)**	**<0.001**	**3.00 (1.05-8.56)**	**0.040**
**Health facility**				
No	Ref.		Ref.	
Yes	**4.85 (2.02-11.63)**	**<0.001**	1.51 (0.26-8.65)	0.645

aOR – Adjusted Odds Ratio, cOR – Crude Odds Ratio, CI – Confidence Interval.

*Boldface type indicates a statistically significant difference at p < 0.05.

In the conditional multivariate model, moderate/high social interaction was associated with higher odds of SARS-CoV-2 infection (aOR 3.00, 95% CI 1.05–8.56). Absence of an underlying condition (aOR 0.25, 95% CI 0.09–0.65) and regular physical activity (aOR 0.18, 95% CI 0.04–0.70) were associated with lower odds. Associations for attending small gatherings and recent travel were elevated in crude analyses but were not statistically significant after adjustment.

## Discussion

Numerous studies have demonstrated factors that facilitate susceptibility or predisposition to COVID-19. There is a clear link between certain pre-existing health conditions and SARS-CoV-2 infection and its adverse prognosis [6]. Among participants with underlying health conditions, hypertension and diabetes were the most commonly reported comorbidities, consistent with findings from previous studies [17–20]. Our study showed a syndemic relationship between SARS-CoV-2 infection and preexisting health conditions. A similar study [20] demonstrated that comorbidity emerged statistically significant with SARS-CoV-2 infection. Additionally, in support, Shahbazi et al reported that SARS-CoV-2 infection was predominant among people who presented with a comorbidity [21]. Furthermore, the outcome of a national study in Saudi Arabia disclosed that a high proportion of the COVID-19 patients had a history of at least one lifelong illness, which seemingly supports this study’s finding [22]*.* On account of the evidence from the above studies, whose findings corroborate those of our study, it is imperative to consider and tailor actions that will safeguard the health needs of persons with a preexisting diagnosis. Individuals with underlying health conditions may experience increased susceptibility to infection and poorer clinical outcomes. Our findings imply that further studies are vital to assess the clinical outcomes of SARS-CoV-2 infection cases in the study jurisdiction.

Epidemiological link is crucial to understanding SARS-CoV-2 infection transmission dynamics, spread and evidence-based control actions. The current study established that attendance at small gatherings showed an increased risk, but was however, not statistically significant in the final multivariate model. Inversely, this finding is on par with Utulu et al. and Graff et al. who reported that attending a mass social gathering was associated with SARS-CoV-2 infection [17,23]. Small gatherings create a perceived sense of safety leading to more relaxed precautions and preventive behaviors such as mask use, physical distancing and attention to ventilation. This false sense of security could facilitate transmission. Moreover, such gatherings typically occur in indoor settings where airflow is limited and individuals remain in proximity for extended periods. In contrast, larger gatherings, which have been more strictly regulated or subject to formal preventive protocols during the pandemic may have entailed greater adherence to public health guidelines. Moderate to high levels of social interaction emerged as an independent predictor of SARS-CoV-2 infection in the adjusted model. Individuals with frequent interpersonal interactions or regular exposure to crowded settings had approximately three times higher odds of infection compared with those reporting lower interaction levels. Frequent social interactions likely increase opportunities for close contact and repeated exposure to infectious respiratory droplets and aerosols, thereby facilitating transmission. Similar observations have been reported in previous studies where increased social mixing patterns and crowd exposure contributed substantially to infection risk [17,23]. This finding underscores the importance of maintaining preventive measures during outbreaks, particularly in social environments where prolonged interpersonal interactions are common.

Regular physical activity has been associated with enhanced immune function, which contributes to a strengthened immune system, potentially reducing the severity and duration of SARS-CoV-2 infection [24,25]*.* Our study uncovered that regular exercise or physical activity decreased the risk of SARS-CoV-2 infection. A similar finding reported by Ho et al. indicated that physical exercise or activity lessened the odds of infection [20]. In support of the above assertions, a study in the United States revealed a negative correlation between physical activity and SARS-CoV-2 infection, which further showed a worse prognosis of death among those who were less physically active [26]*.* Moreover, according to Zhang et al.*,* among adults aged between 40 and 69 years, those who were physically active were less likely to be confirmed SARS-CoV-2 seropositive [27]. However, in dissonance with the aforementioned findings, Yeo established that physical activity was not associated with the risk of SARS-CoV-2 infection [28]. Similarly, it was also conversely reported that physical activity had no protective association between SARS-CoV-2 infection and its related symptoms [29]. Physical inactivity predisposes to longstanding diseases like hypertension and diabetes, which are established comorbidities that increase SARS-CoV-2-infection susceptibility or severity and point out the significance of regular physical activity and exercise in SARS-CoV-2 infection prevention [29]*.* Although it is widely asserted that regular physical activity is a non-pharmacological intervention against SARS-CoV-2 infection and could help alleviate the symptoms and severity [30], there is uncertainty regarding the effects on SARS-CoV-2-infected individuals, despite some evidence to the effect that physical exercise improves respiratory and physical health.

### Strengths and limitations

This study provides localized evidence to inform tailored public health interventions and contributes to the broader understanding of SARS-CoV-2 epidemiology. A major strength of this study is the use of a matched case-control design, which controlled for major confounders such as age and sex, thereby enhancing the internal validity of the observed associations. Nonetheless, several limitations should be acknowledged. First, recall bias was a potential limitation due to the retrospective nature of data collection, which occurred approximately three years after the initial SARS-CoV-2 testing period (2020–2021). Participants may have had difficulty accurately recalling past exposures or conditions. To minimize this, we used structured questionnaires, restricted recall to major exposures, and cross-validated responses with available surveillance records where possible. However, residual recall bias may have affected the measurement of exposures.

Second, the study may have been subject to social desirability bias, particularly in the self-reporting of preventive behaviors. Cases, for instance, may have overreported adherence to recommended public health measures, potentially leading to attenuated or biased associations. Finally, incomplete information such as missing contact details in the surveillance line list, may have introduced selection bias during the sampling process, which could affect the generalizability of the findings.

## Conclusion

This study identified moderate to high levels of social interaction as an important factor associated with increased odds of SARS-CoV-2 infection, while regular physical activity and absence of underlying health conditions were protective. These findings emphasize the role of behavioral and individual health factors in influencing susceptibility to infection. Public health interventions should prioritize strengthening community awareness regarding safe social practices and promotion of healthy lifestyles, particularly regular physical activity. Furthermore, targeted preventive strategies for individuals with underlying health conditions may help reduce vulnerability during future outbreaks. Lessons from this study provide important insights for strengthening preparedness and response strategies against future pandemics in Ghana and similar settings. Further research is needed to explore the long-term effects of these risk and protective factors on SARS-CoV-2 infection outcomes.

## Abbreviations

COVID-19: Coronavirus Disease
NHIS: National Health Insurance Scheme
RT-PCR: Reverse transcription polymerase chain reaction
SARS-CoV-2: Severe Acute Respiratory Syndrome Coronavirus-2
WHO: World Health Organization
